# Civilization

**Published:** 1869-11

**Authors:** 


### CIVILIZATION.

At the recent meeting at Exeter of the British Association for the Advancement of Science, Sir George Grey remarked that the highest state of civilization was the highest development of Christianity—the unselfishness of man, and regard for the welfare of his fellow-man—and he believed that this virtue had in every case been introduced among the various races of mankind by some race who claimed (he would not say rightly or wrongly) by inspiration to have received a knowledge of its truths. In every case where people did recognize duties of that kind, they affirmed that they had received the knowledge in this way. He had been much among savages, but had never seen any tendency in them to advance in the civilization of which he had spoken, or in the arts that were beneficial to mankind generally.

